# Emergence of a Novel Recombinant Pseudorabies Virus Derived From the Field Virus and Its Attenuated Vaccine in China

**DOI:** 10.3389/fvets.2022.872002

**Published:** 2022-04-26

**Authors:** Lei Tan, Jun Yao, Lei Lei, Kaiwen Xu, Fan Liao, Shibiao Yang, Lincheng Yang, Xianghua Shu, Deyong Duan, Aibing Wang

**Affiliations:** ^1^Lab of Animal Disease Prevention and Control and Animal Model, Hunan Provincial Key Laboratory of Protein Engineering in Animal Vaccines, College of Veterinary Medicine, Hunan Agricultural University (HUNAU), Changsha, China; ^2^Yunnan Tropical and Subtropical Animal Virus Diseases Laboratory, Yunnan Animal Science and Veterinary Institute, Kunming, China; ^3^College of Animal Medicine, Yunnan Agricultural University, Kunming, China; ^4^PCB Biotechnology LLC, Rockville, MD, United States

**Keywords:** pseudorabies virus, natural recombination, genetic analysis, pathogenicity, China

## Abstract

The occurrence of pseudorabies (PR) caused by the PR virus (PRV) causes huge economic losses to the pig industry in China. Moreover, the potential threat of PRV to humans' health has received wide attention recently. The prevalence of two PRV genotypes and the application of their corresponding live attenuated vaccines increase the recombination possibility. In the present study, a novel recombinant PRV strain designed as HN-2019 was isolated from one sick piglet in Hunan province, China, its genetic features and pathogenicity were further investigated. The results showed that the glycoprotein E (*gE*) and *gG* genes of the HN-2019 strain displayed higher nucleotide homology with PRV classical strains (such as Ea and Fa) compared to others. However, its *TK* gene with continuous nucleotide deletions shared 100% nucleotide identity with the HB-98 vaccine strain, which was derived from the Ea strain. Moreover, the HN-2019 strain exhibited similar growth characteristics to that of the Ea strain, but its pathogenicity in mice was significantly lower than the latter one. The results above suggested that a naturally recombinant event might occur in the genome of the HN-2019 strain between the PRV classical strain and the HB-98 vaccine strain, which will provide useful guidelines for PRV vaccine design in the future.

## Introduction

Pseudorabies virus (PRV) belongs to the genus *Varicellovirus* and is also called Suid herpesvirus (SuHV-1) or Aujeszky's disease virus (ADV). Pseudorabies (PR) caused by PRV has been considered infectious and fatal to pigs, the clinical symptoms of which are mainly characterized by vomiting, diarrhea, and severe neurological disease with high mortality in newborn piglets, abortion in sows, and dyspnea in fattening pigs (1, 2). Moreover, PRV can also infect many other mammals, such as dogs, wolves, minks, foxes, sheep, goats, cattle, and bears (3). Recently, more than 22 clinical cases of PR in humans have so far been documented in China (4). In particular, a variant PRV strain has successfully been isolated from one human encephalitis case (5), indicating the potential threat of PRV to human beings.

Pseudorabies virus is a double-stranded linear DNA virus with a nearly 150 kb genome encoding more than 70 proteins (6). Among numerous PRV protein-encoding genes, the glycoprotein E (*gE*), *gI*, and *TK* genes are essential to viral pathogenicity, while deletion(s) of these genes do not alter viral immunogenicity (7). PRV strains can be divided into two genotypes according to their genetic characteristics, the genotype I is mainly composed of PRV strains prevalent in Europe and USA, including the Bartha strain, while most Chinese strains (variant and classical PRV strains) belong to the genotype II group (4). To effectively control PR in China, various types of vaccines mainly including inactivated and live attenuated vaccines have been developed and widely applied in the pig industry (8).

The first live-attenuated PRV vaccine (Bartha-K61 strain) was introduced in Hungary in the 1970s and has widely been applied in China for decades (9). Since 2011, the diseases caused by PRV variants have frequently occurred in many Bartha-K61-immunized pig herds in China, suggesting that the Bartha-K61 vaccine failed to provide full protection against PRV variants (9, 10). Subsequently, two live attenuated PRV vaccines [HB-98 (Ea-Δ*gE/gG/TK*) and SA215 (Fa-Δ*gE/gI/TK*)] derived from classical PRV strains (Ea and Fa) were licensed in China, the application of which has greatly contributed to the control of PR in China (11, 12).

Natural recombination is considered a key factor accelerating virus genomic evolution, which may affect viral pathogenicity (13). In this regard, the occurrences of recombinant PRV strains generated from different PRV genotypes (genotype I and genotype II) have been documented in China recently (14, 15). However, the natural recombinant event of the PRV genome from the field PRV strain and its derived live attenuated vaccine has not been reported yet. In this study, a novel naturally occurring recombinant PRV strain HN-2019 was identified, and its growth characteristics *in vitro* and pathogenicity in mice were further investigated.

## Materials and Methods

### Sample Collection and Virus Isolation

In April 2019, one case of PR was documented on a pig farm in Hunan province of China, where only piglets displayed mild clinical symptoms, including high fever, and mild respiratory disorders with low mortality. Tissue samples (including tonsil and mandible lymph glands) were obtained from one sick piglet to identify the causative agent(s), homogenized with DMEM medium, and subjected to three freezing-thawing cycles. All samples were identified as PRV-positive, but negative for other pathogens (porcine circovirus type 2, porcine reproductive and respiratory syndrome virus, classical swine fever virus, bacteria, etc.) by PCR/RT-PCR (data not shown).

Subsequently, porcine kidney (PK15) cells were inoculated with the supernatant of the PRV-positive tonsil sample for 2 h, then cultured in DMEM supplemented with 5% fetal bovine serum (FBS), 100 IU/ml penicillin, and 100 μg/ml streptomycin at 37°C under humid 5% CO_2_ atmosphere. The culture media containing viruses were harvested when nearly 80% of cells showed obvious cytopathic effects cytopathic effects (CPEs) and further purified *via* plaque purification as described previously (16). An indirect immunofluorescent assay (IFA) was conducted to detect the presence of the infectious viruses using a PRV-specific gE antibody, which was a gift from professor Ping Jiang at Nanjing Agricultural University.

### Sequencing and Phylogenetic Analysis

Total genomic DNAs were extracted from cell cultures containing viruses using commercial kits (Takara, Dalian, China). The full-length *gE, TK*, and *gG* genes were amplified by PCR as described previously (17), the specific primers were listed in Supplementary Table S1. Purified PCR fragments were sent for sequencing (TSINGKE, Changsha, China) and submitted to the GenBank database.

The *gE, TK*, and *gG* gene sequences of 17 representative PRV reference strains were downloaded from the GenBank database (Supplementary Table S2). The genetic characteristics of these nucleotide sequences were analyzed using DNAStar version 7.10 (Lasergene DNAStar software), phylogenetic trees based on the *gE, gG*, and *TK* genes were reconstructed using MEGA7.0 software with the maximum likelihood (ML) method [time-reversible (GTR) model; 1,000 bootstrap replicates).

### Growth Characteristics of HN-2019 *in vitro*

To further investigate the growth characteristics of HN-2019, the HB-98 vaccine, and Ea strains were set as control groups. A monolayer of PK15 cells was infected with different PRV strains at an MOI of 1.0 for 1 h, respectively. Subsequently, the cells were washed and incubated with DMEM containing 2% FBS. The supernatant and cells were harvested every 12 h post-infection. After being freeze-thawed three times, the viruses were titrated by the Reed-Muench method in PK15 cells, which was designed as a 50% tissue culture infectious dose per ml (TCID_50_/ml).

### Pathogenicity Analysis in Mice

Six-week-old healthy female Kunming mice were purchased from Hunan SJA Laboratory Animal Co., Ltd. Six mice in each group were challenged with 10^4^ TCID_50_ of HN-2019, Ea, or HB-98 vaccine strain *via* hind footpad injection. Mice in the placebo group were injected with an equal volume of DMEM. The healthy conditions of mice in different groups were monitored two times per day, and the survival curves of mice were generated using GraphPad Prism 8 software (GraphPad Software, La Jolla, CA, USA). All survival mice were euthanized at 15 days post-inoculation, viral loads of different tissue samples (brain, lung, liver, and spleen) in three groups were determined *via* a verified real-time PCR assay as described previously (18).

### Statistical Analysis

All experiments were conducted in a triple, the statistical difference between different groups was analyzed with a *t*-test using GraphPad Prism 8 software. The data were presented as mean ± *SD*. Differences with *P*-value < 0.05 were determined as statistically significant.

## Results

### Isolation and Biological Features of HN-2019 Strain

**Figure 1:**
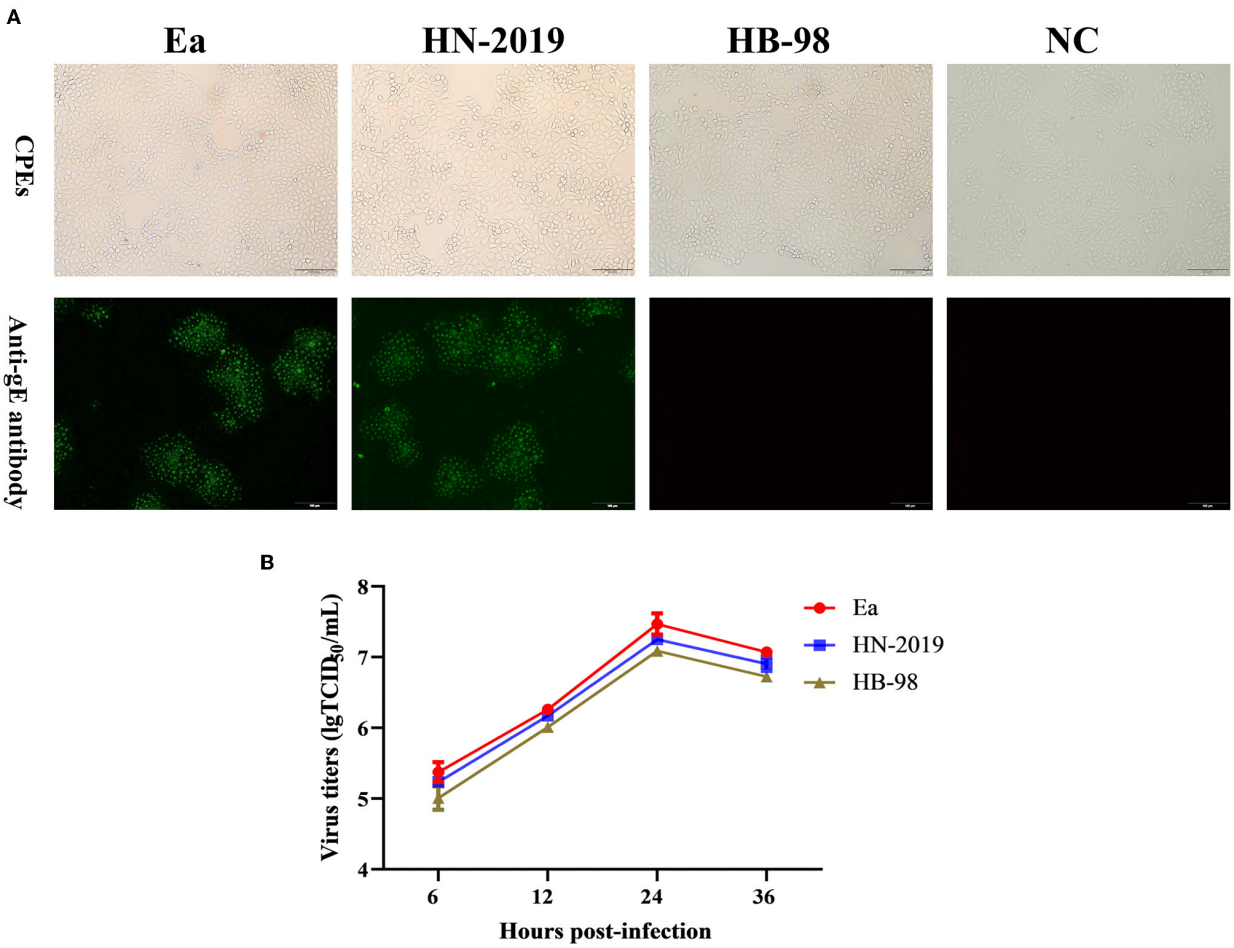
The biological properties of a novel recombinant PRV strain (HN-2019), Ea, or HB-98 vaccine strains. The cytopathic effects (CPEs) in porcine kidney (PK15) cells infected with HN-2019 were characterized by rounded and floated cells, with the CPEs in HB-98 or Ea-infected PK15 cells set as positive controls **(A**, upper panels). Indirect immunofluorescent assay for detecting the gE protein in PK15 cells infected with HN-2019, Ea, or HB-98 vaccine strains, respectively (Scale bar = 100 μm) **(A**, lower panels). One-step growth curves of three pseudorabies virus (PRV) strains in PK15 cells at an MOI of 1. The viral titers of them were determined by TCID_50_/ml **(B)**.

The PRV strain designed as HN-2019 was successfully isolated in PK15 cells with the observation of typical PR-specific CPEs, and the CPEs caused by the HN-2019 strain were similar to those of Ea and HB-98 vaccine strains (Figure 1A). The presence of infectious viruses was further validated *via* IFA (Figure 1A). Moreover, one-step growth curve experiments revealed that the growth kinetics of the HN-2019 strain were similar to those of Ea and HB-98 vaccine strains, while the HN-2019 strain replicated slightly faster than the HB-98 vaccine strain but lower than the Ea strain in PK15 cells (Figure 1B).

### Genetic Features and Phylogenetic Analysis of HN-2019 Strain

**Figure 2:**
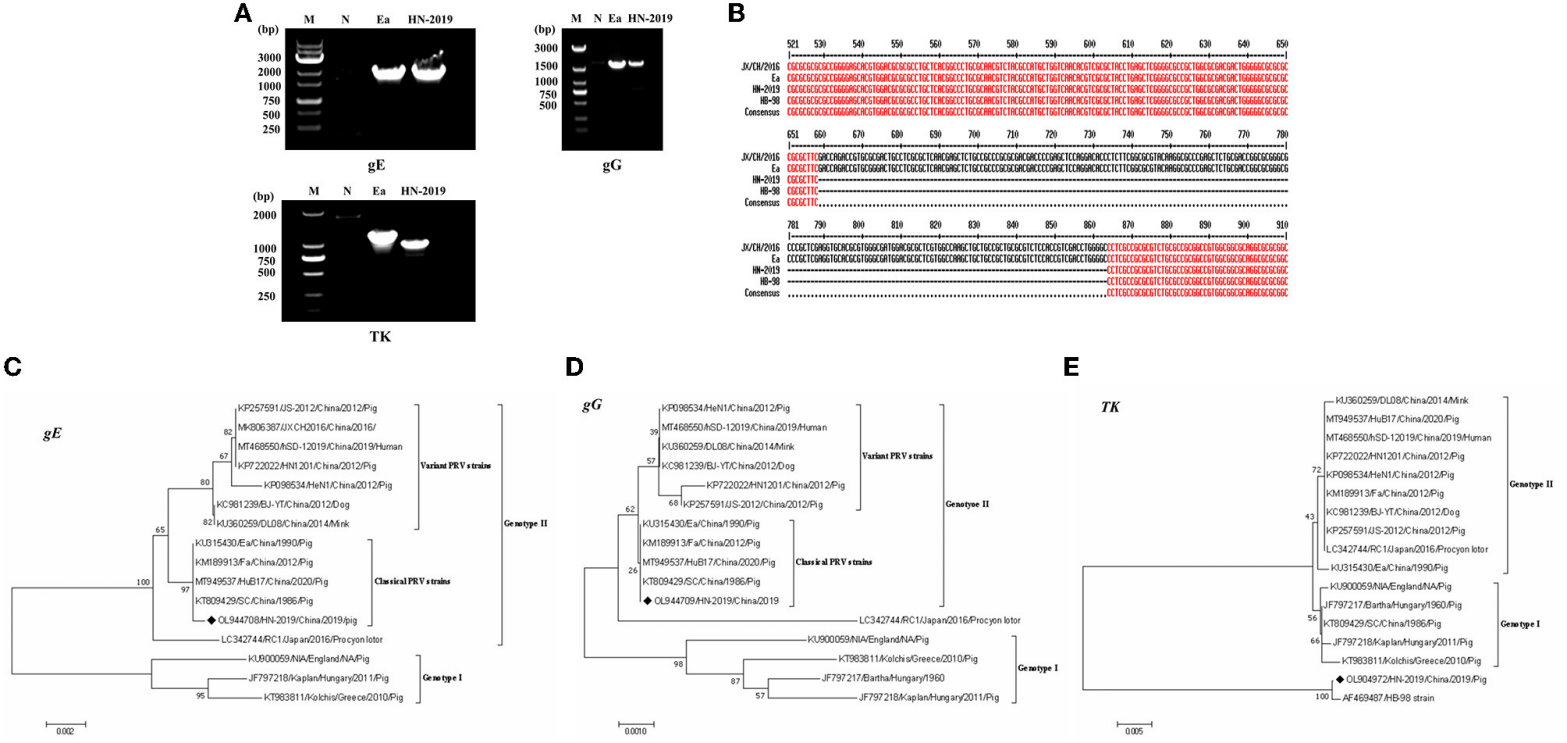
Detection of the PRV *gE, gG*, or *TK* genes by PCR. Wild PRV (Ea) strain and HN-2019 strain genomes were extracted and their *gE, gG*, and *TK* genes were amplified by PCR using three pairs of primers. The altered size of the PRV *TK* gene of HN-2019 **(A)**. Nucleotide sequence alignment of the *TK* genes of JX/CH/2016, HN-2019, Ea, and HB-98 vaccine strains **(B)**. Phylogenetic trees based on the *gE, TK*, or *gG* nucleotide sequences were generated using MEGA7 software with the ML method [time-reversible (GTR) model; 1,000 bootstrap replicates] **(C–E)**.

The full-length *gE* (Accession No. OL944708), *TK* (Accession No. OL904972), and *gG* (Accession No. OL944709) gene sequences of the HN-2019 strain were amplified *via* PCR (Figure 2A). Sequence analysis showed that the *gE* and *gG* genes shared over 99.5 and 100.0% nucleotide sequence homology with classical PRV strains (including Ea and Fa), respectively. Interestingly, a continuous deletion of 205 nucleotides was observed in the *TK* gene of HN-2019 compared to other PRV strains, while this genetic feature also existed in the HB-98 vaccine strain, which was derived from the Ea strain and has been widely applied in Chinese pig populations (Figure 2B).

Phylogenetic trees based on the *gE, gG*, and *TK* genes of PRV strains were reconstructed using the ML method. The phylogenetic positions of the HN-2019 *gE* and *gG* genes were closer to those of classical PRV strains compared with others (Figures 2C,D), but the *TK* genes of HN-2019 and HB-98 vaccine strains were classified into the same branch (Figure 2E), indicating that HN-2019 might be a natural recombinant from the classical PRV strain (such as Ea) and HB-98 vaccine strain.

### Altered Virulence of PRV HN-2019 Compared to Ea Strain in Mice

**Figure 3:**
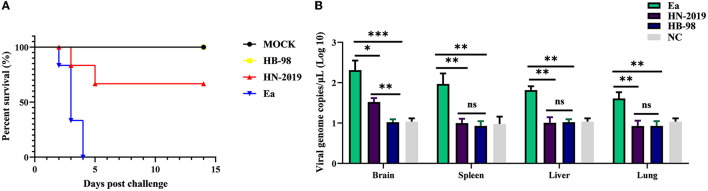
Kaplan-Meier survival curves of mice challenged with 10^4^ TCID_50_ of HN-2019, Ea, or HB-98 vaccine strains **(A)**. Viral copies of brain, liver, lung, and spleen samples in each group (*n* = 3) were determined by real-time PCR assays **(B)**. Significant differences were considered when *P* < 0.05 (*), *P* < 0.01 (**), and *P* < 0.001 (***).

To investigate the pathogenicity of the HN-2019 strain *in vivo*, mice were challenged with 10^4^ TCID_50_ of HN-2019, HB-98, or Ea strain by hind footpad injection. The results showed that incubation with a high dose of Ea strain was lethal to all mice, with the death rate reaching 100% (6/6), while the mortalities of HN-2019 and HB-98 strains at the same dose were 33.33% (2/6) and 0% (0/6), respectively (Figure 3A). Furthermore, the viral loads of different tissue samples in the Ea-infected group were higher than the corresponding samples in the HN-2019-infected group (Figure 3B). This data manifested that the virulence of the HN-2019 strain in mice was lower than that of the Ea strain while higher than the HB-98 vaccine strain.

## Discussion

Pseudorabies virus has been widely prevalent in Chinese pig populations, meanwhile, the capability of PRV infecting various species of animals, even human beings, truly raises widespread concerns (5, 19). Though the PRV Bartha-K61 vaccine has been developed and used for decades, its shortcomings are gradually appearing mainly owing to long-term application. Especially, growing evidence showed that the Bartha-K61 vaccine could not provide complete protection against the emerging variant PRV strains (9, 10). Consequently, besides Bartha-K61, another two HB-98, and SA215 strains based on live attenuated PRV vaccines have been developed and applied to efficiently control PR in China. In spite of the relative conservativeness of the PRV genomic DNA, novel PRV strains originated from the natural recombination of different PRV genotypes or live attenuated vaccines and wild PRV strains have been documented in China recently (14, 15, 20).

**Figure 4:**
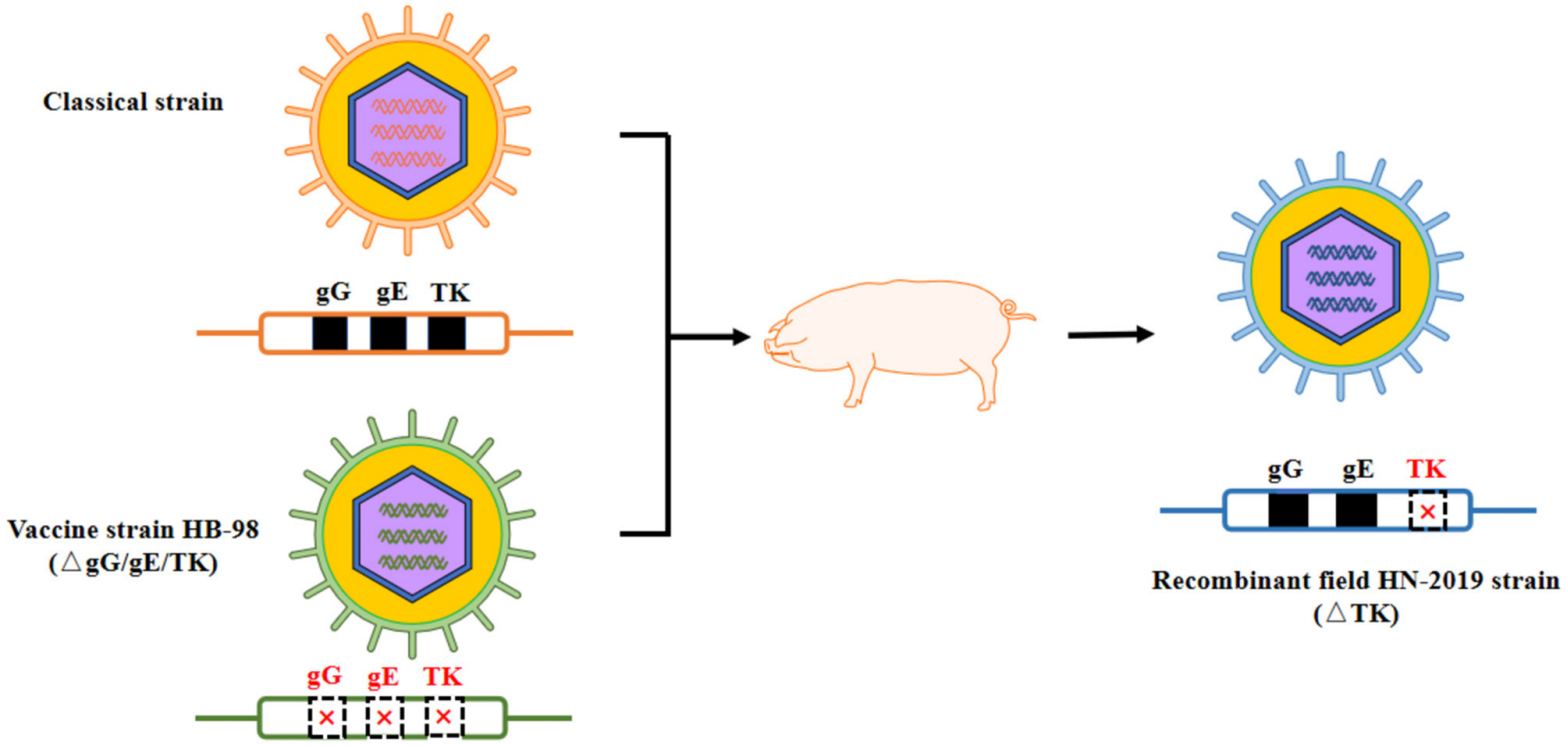
Proposed model of genomic recombination of HN-2019 strain. HB-98-vaccinated pigs were infected with classical PRV strain. During the replication of two PRV strains in pigs, HN-2019 with *TK* gene deletion was generated.

In this study, a novel field PRV strain named HN-2019 strain was successfully isolated in Hunan province, China, and its genetic features and replication characteristics were further investigated. Significantly, this newly isolated HN-2019 strain was speculated to derive from a recombinant event occurring between the field PRV strain and its derived live attenuated vaccine strain. The supportive evidence included: firstly, the HN-2019 strain presented similar growth features as HB-98 and Ea strains *in vitro*; secondly, the genetic analysis of the HN-2019 *gE, gG*, and *TK* genes revealed that the former two genes had nearly 100% nucleotide sequence identity with those of classical PRV strains, while the homology of the HN-2019 *TK* gene with HB-98 vaccine strain reached to 100%, similarly with a continuous deletion of 205 nucleotides within them compared with those of others; thirdly, the *in vivo* virulence of HN-2019 strain was closer to that of HB-98 rather than Ea strain. The large PRV DNA genome allows to delete some virulent gene (s) such as *gE, gI*, and *TK* genes in the absence of an impact on its viral replication (21), together with the fact that the HB-98 vaccine with triple deleted genes (i.e., *gE*/*gG*/*TK*) is derived from PRV Ea strain, therefore, it is logical to consider that the HN-2019 strain may be a novel recombinant one generated from the classical strain and HB-98 vaccine strain (Figure 4).

Since the *TK* gene is responsible for PRV virulence, accordingly, *TK*-negative PRV isolate was highly attenuated in different animal models compared with the wild strains (22, 23). In accordance with this cognition, our results also confirmed that challenge with Ea strain led to a high fatality rate in mice, while the efficiency of similar dose *TK*-deficient PRV strain (HN-2019) exposure was greatly compromised (100% vs. 33.33%). Despite this, diseased piglets infected by this HN-2019 strain displayed mildly typical PR clinical symptoms, suggesting that the occurrence of the HN-2019 strain also posed a threat to pig populations, which should not be ignored.

PRV strains prevalent worldwide could be classified into two genotypes, while growing evidence indicated the occurrence of recombinant events in the same genotype such as classical and variant PRV strains in genotype II (24), even between these two genotypes like Bartha-K61 vaccine (genotype I) and variant PRV strains (genotype II) (14). However, a report of the recombination occurring between the field PRV and its derived vaccine strains has not been presented prior to this study. Therefore, the current report could be considered to fill in this gap, suggesting that the wide application of live attenuated vaccines might increase the probability of recombination between the vaccine strain and other field strains, implying that the development of safer and more effective vaccines are urgently required for the control and eradication of PR in the future (25).

## Conclusion

In summary, a novel PRV strain designed as HN-2019 was identified to derive from the recombination between classical PRV (such as Ea) and HB-98 vaccine strains. Notably, though the biological properties of this HN-2019 strain were similar to those of the parental strains, its virulence in mice lay between the two parental strains. Therefore, this observation, together with previous reports, highlights the significance of continuous monitoring of recombinant events in PRV strains, which is essential for vaccine design and the eradication of PR in China.

## Data Availability Statement

The datasets presented in this study can be found in online repositories. The names of the repository/repositories and accession number(s) can be found in the article/Supplementary Material.

## Ethics Statement

All animal experiments were conducted according to the rules of Animal Ethics Committee of the Hunan Agricultural University, Changsha, China (43321503), and the mice were housed in the animal facility of Hunan Agricultural University (Changsha, Hunan, China).

## Author Contributions

AW and LT: designed the experiments. LT, JY, LL, KX, FL, SY, XS, LY, DD, and AW: investigation and methodology. LT, JY, and LL: formal analysis. AW, LT, and JY: writing-original draft preparation. AW and JY: funding. All authors have read and agreed to the published version of the final manuscript.

## Funding

This work was supported by the General Program of the National Natural Science Foundation of China (Grants No. 31571432/31802252), the National Key Research and Development Program of China (No. 2017YFD0501800), the Major Specialized Projects of Yunnan Science and Technology (No. 202102AE090007), the Hunan Provincial Natural Science Foundation of China (2020JJ4041), the Postgraduate Scientific Research Innovation Project of Hunan Province (CX20200659), Support was also provided by Furong Scholar funding to AW.

## Conflict of Interest

AW was employed by PCB Biotechnology LLC. The remaining authors declare that the research was conducted in the absence of any commercial or financial relationships that could be construed as a potential conflict of interest.

## Publisher's Note

All claims expressed in this article are solely those of the authors and do not necessarily represent those of their affiliated organizations, or those of the publisher, the editors and the reviewers. Any product that may be evaluated in this article, or claim that may be made by its manufacturer, is not guaranteed or endorsed by the publisher.
