# Supplementary material for: Emergence of a Novel Recombinant Pseudorabies Virus Derived From the Field Virus and Its Attenuated Vaccine in China
Source: Front Vet Sci. 2022 Apr 26;9:872002. doi: 10.3389/fvets.2022.872002 (PMC9087331; doi:10.3389/fvets.2022.872002)
Supplement: Supplementary file 1 [file Table_1.DOC]

| Primer | Sequence 5’-3’ | Binding position | Length | Purpose | Reference sequence |
| --- | --- | --- | --- | --- | --- |
| PRV-*TK*-F | TCGTAGAAGCGGTTGTGGC | 59458-60591 | 1134 | Sequencing | [MK806387](https://www.ncbi.nlm.nih.gov/nucleotide/MK806387.1?report=genbank&log$=nuclalign&blast_rank=1&RID=ZU03AUKV016) |
| PRV-*TK*-R | GGCAAACTTTATTGGGATGA |
| PRV-*gE*-F | TTTGTGGGTGGCGTTTTATCTC | 123287-125244 | 1958 | Sequencing | [MK806387](https://www.ncbi.nlm.nih.gov/nucleotide/MK806387.1?report=genbank&log$=nuclalign&blast_rank=1&RID=ZU03AUKV016) |
| PRV-*gE*-R | AGCAGTCCGAGTCGTCCTGG |
| PRV-*gG*-F | ATGAGATACTCAACTTTGGAATGT | 119160-120855 | 1695 | Sequencing | [MK806387](https://www.ncbi.nlm.nih.gov/nucleotide/MK806387.1?report=genbank&log$=nuclalign&blast_rank=1&RID=ZU03AUKV016) |
| PRV-*gG*-R | TAAGCAAGGCCGTACGCAAA |

**Supplementary table 1** Primers used for PCR amplification in this study
