# Supplementary material for: Emergence of a Novel Recombinant Pseudorabies Virus Derived From the Field Virus and Its Attenuated Vaccine in China
Source: Front Vet Sci. 2022 Apr 26;9:872002. doi: 10.3389/fvets.2022.872002 (PMC9087331; doi:10.3389/fvets.2022.872002)
Supplement: Supplementary file 2 [file Table_2.DOC]

| Strain | GenBank accession | Country | Isolate date | Genotype (I/II) | Species |
| --- | --- | --- | --- | --- | --- |
| Bartha | JF797217 | Hungary | 1960 | I | Pig |
| Kaplan | JF797218 | Hungary | 2011 | I | Pig |
| Kolchis | KT983811 | Greece | 2010 | I | Pig |
| Ea | KU315430 | China | 1990 | II | Pig |
| Fa | KM189913 | China | 2012 | II | Pig |
| SC | KT809429 | China | 1986 | II | Pig |
| HeN1 | KP098534 | China | 2012 | II | Pig |
| HN1201 | KP722022 | China | 2012 | II | Pig |
| JS-2012 | KP257591 | China | 2012 | II | Pig |
| HuB17 | MT949537 | China | 2020 | II | Pig |
| JX/CH/2016 | MK806387 | China | 2016 | II | Pig |
| BJ/YT | KC981239 | China | 2012 | I | Dog |
| DL/08 | KU360259 | China | 2014 | II | Mink |
| hSD-1/2019 | MT468550 | China | 2019 | II | Human |
| HN1201 | KP722022 | China | 2012 | II | Pig |
| RC1 | LC342744 | Japan | 2016 | II | Procyon lotor |
| NIA3 | KU900059 | England | NA | I | Pig |

**Supplementary table 2** Information of PRV reference strains
